# Pulmonary Telerehabilitation and Telemonitoring for Patients with Chronic Obstructive Pulmonary Disease: A Single-Arm Pilot Feasibility Study

**DOI:** 10.3390/jcm15135292

**Published:** 2026-07-07

**Authors:** Matías Otto-Yáñez, Rodrigo Torres-Castro, Lea Soliman, Onah Chiemelie, Sandra C. Webber, Renata Mancopes, Antonio Sarmento, Diana C. Sanchez-Ramirez

**Affiliations:** 1 Grupo de Investigación en Salud, Funcionalidad y Actividad Física (GISFAF), Kinesiología, Facultad de Ciencias de la Salud, Universidad Autónoma de Chile, Santiago 7500912, Chile; matias.otto@uautonoma.cl; 2 Department of Physical Therapy, Faculty of Medicine, University of Chile, Santiago 8380453, Chile; rodritorres@uchile.cl; 3 Department of Respiratory Therapy, Rady Faculty of Health Sciences, University of Manitoba, Winnipeg, MB R3E 0T6, Canada; lea.soliman@umanitoba.ca (L.S.); onahc@myumanitoba.ca (O.C.); antonio_sarmento_@hotmail.com (A.S.); 4 Department of Physical Therapy, Rady Faculty of Health Sciences, University of Manitoba, Winnipeg, MB R3E 0T6, Canada; sandra.webber@umanitoba.ca; 5 KITE Toronto Rehabilitation Institute, University Health Network, Toronto, ON M5G 2A2, Canada; renata.mancopes@uhn.ca

**Keywords:** COPD, pulmonary rehabilitation, telerehabilitation, telemonitoring, feasibility, exercise training

## Abstract

**Background**: Pulmonary rehabilitation is effective for people with chronic obstructive pulmonary disease (COPD), but access remains limited. Telerehabilitation with telemonitoring may improve access to home-based care; however, feasibility data are currently scarce. This study assessed the feasibility, acceptability, and occurrence of adverse effects in a videoconference-based pulmonary telerehabilitation program supported by wearable devices in individuals with COPD. **Methods**: Our one-group pre-post feasibility study evaluated an 8-week intervention comprising two supervised exercise sessions and one education session per week. Telemonitoring included wearable-device data capture (heart rate, peripheral oxygen saturation, step count) and symptom severity reporting. Feasibility outcomes included recruitment rate, study completion, dropout rate, session attendance, data-submission compliance, adverse events, and participant satisfaction. Exploratory clinical outcomes were assessed pre- and post-intervention. **Results**: Of 32 eligible individuals, 15 consented and attended baseline assessment (recruitment rate: 47%), and nine completed the study (completion: 60%; dropout: 40%). Among completers, median age was 67 years (interquartile range [IQR] 62–73), and seven (78%) were women. Mean session attendance was 94 ± 6.6%. Data submission rates averaged 86 ± 7.7% for the O2 ring and 97 ± 6.4% for symptom severity reporting, while smartwatch data were submitted by all participants (100%). No adverse events were reported, and participant satisfaction was high. No statistically significant pre-post changes were observed in clinical outcomes. Mean 6-min walk test distance increased by 29 m, a potentially clinically relevant but exploratory finding. **Conclusions**: A videoconference-based pulmonary telerehabilitation program supported by wearable devices appears to be operationally deliverable among participants who remained engaged, with no adverse events observed in this small, selected group of participants with COPD. Findings should be interpreted cautiously due to the small sample size, one-group design, and attrition.

## 1. Introduction

Chronic obstructive pulmonary disease (COPD) remains a major cause of morbidity, disability, and healthcare utilization worldwide. Its burden is expected to continue increasing over the coming decades, with an estimated of 600 million of people living with COPD globally by 2050 [1]. In parallel, recent Global Initiative for Chronic Obstructive Lung Disease (GOLD) updates continue to emphasize that COPD management must go beyond pharmacological treatment and include evidence-based non-pharmacological strategies to reduce symptoms, improve function, and support long-term disease self-management [2].

Pulmonary rehabilitation (PR) is one of the most effective non-pharmacological interventions for COPD [3]. Contemporary statements and guidelines define PR as a comprehensive, patient-tailored intervention that integrates exercise training, education, and behavior change to improve the physical and psychological condition of people with chronic respiratory disease and to promote long-term adherence to health-enhancing behaviors [4,5]. High-quality evidence has consistently shown that PR improves dyspnea, fatigue, exercise capacity, health-related quality of life, and perceived control over disease in people with COPD [6]. In addition, PR delivered after COPD exacerbation has been associated with important benefits in exercise performance and health status, and guideline panels now strongly recommend PR both for stable COPD and after hospitalization for exacerbation [7,8,9].

Despite these well-established benefits, access to PR remains poor [10]. Underuse reflects a complex combination of health-system, clinician, and patient-level barriers, including limited program availability, long travel distances, transportation difficulties, competing responsibilities, inadequate referral processes, and low awareness of PR content and benefits [10,11]. Environmental context, personal knowledge, and beliefs are among the most influential determinants of referral, uptake, attendance, and completion [12,13,14]. Recent reviews have further highlighted that, globally, only a small minority of eligible people with chronic lung disease actually access PR, emphasizing the need for scalable alternative delivery models [12,13,14].

In Manitoba, PR is delivered in-person through a publicly funded Winnipeg Regional Health Authority (WRHA) program operating across three sites in Winnipeg [15]. Outside of Winnipeg, there are currently no formal PR programs available, posing a substantial challenge to equitable access to care. This gap is particularly relevant given that a considerable proportion of the provincial population resides in rural and remote communities, where geographic distance and travel demands further constrain participation in centre-based services. In a previous real-world evaluation of this program, 1085 patients with chronic respiratory diseases participated between 2016 and 2019, and 63% completed the intervention. Patients who completed the program were older and had better baseline outcomes than those who did not complete it, highlighting persistent barriers to uptake and completion and supporting the need to explore alternative PR delivery models that may improve accessibility and adherence [15].

Telerehabilitation has emerged as one such alternative. A Cochrane review and subsequent meta-analyses suggest that telerehabilitation can achieve clinically relevant gains in exercise capacity, dyspnea, and quality of life, while offering safety and adherence profiles that are broadly comparable to conventional center-based programs [16,17]. Randomized trials have also shown that remotely delivered rehabilitation may be equivalent to center-based PR for selected outcomes and may support broader access for patients who cannot attend in-person services [18,19,20]. At the same time, the literature remains heterogeneous with respect to intervention structure, level of supervision, technologies used, and outcome reporting, which limits implementation in routine practice [9,17].

Telemonitoring and wearable devices may further strengthen remote PR models by enabling continuous or near-continuous capture of physiological and symptom data, supporting safety surveillance, patient engagement, and individualized feedback [21]. However, current evidence suggests that although wearable-enabled interventions can improve physical activity and some functional outcomes, their clinical value is variable, often short-lived, and greater when devices are embedded within broader behavioral or rehabilitation programs rather than used in isolation [22]. Reviews of remote patient monitoring in COPD also emphasize ongoing challenges related to integration, standardization, and translation into real-world care pathways [21,23].

Therefore, the present study aimed to evaluate the feasibility of an 8-week pulmonary telerehabilitation program supported by commercial wearable telemonitoring devices in people with COPD, and to explore its effects on dyspnea, fatigue, exercise capacity, self-efficacy, and health-related quality of life.

## 2. Methods

### 2.1. Study Design and Ethics

This study was designed as a single-arm pre-post pilot feasibility study. Reporting was guided by the STROBE statement [24], using the items considered most applicable to this design. The protocol was approved by the University of Manitoba Research Ethics Board (HS25892 B2023:030), and all participants provided written informed consent before enrolment.

### 2.2. Participants and Recruitment

A convenience sample of adults with a confirmed diagnosis of COPD living in Manitoba (Canada) was recruited through public advertising, patient support groups, and referrals from healthcare providers. No formal sample size calculation was performed, as this was a feasibility study not designed to test effectiveness hypotheses. A target of 15 participants was considered appropriate to estimate key feasibility parameters—including recruitment rate, completion, attendance, and compliance—with sufficient precision to inform the design of a future trial, consistent with recommendations for pilot and feasibility research [25,26]. Eligible participants were required to have access to a laptop, smartphone or tablet, and home internet service. A brief clinical safety screening was used to identify potential contraindications to remotely supervised exercise, including recent exacerbation or hospitalization, cardiovascular conditions, pre-existing conditions that limit physical activity, fall risk, and sensory impairments. Those with acute exacerbation of COPD, history of neurological disease or mental illness, inability to ambulate independently without supervision, and inability to complete basic tasks using a smartphone or tablet were excluded.

### 2.3. Intake Assessment and Orientation

Participants attended an initial in-person visit of approximately 2 h at the RespirabilityLab (Riverview Health Centre, Winnipeg, MB, Canada). During this visit, the intervention was explained, and baseline assessments were completed. Based on this assessment, a member of the research team provided individualized safety recommendations, including personalized guidance on target exercise intensity, maximum heart rate, and minimum oxygen saturation (SpO_2_) following established standards [27,28]. Two participants were receiving supplemental oxygen therapy during the study. They were instructed to continue using oxygen according to their usual prescribed recommendations, including any exercise-specific flow rate adjustments previously advised by their healthcare providers.

They also received training in using Zoom^®^ version 5.17.11 (Zoom Video Conferencing Software Inc., San Jose, CA, USA), a symptom-reporting Labfront Companion application (Kiipo Co., d/b/a Labfront, Sharon, MA, USA), a Garmin Venu Sq 2 smartwatch (Garmin International, Inc., Olathe, KS, USA), and a smart O2 ring (LOOKEE Tech, Vancouver, BC, Canada), along with their associated applications. The O2 ring allowed for the setting of individualized safety thresholds for heart rate (60–80% of age-predicted maximum heart rate [28]) and SpO_2_ (≥88%) and activated a vibration cue when the set limits were reached, prompting users to consider stopping the activity.

### 2.4. Intervention

The intervention lasted eight weeks and combined synchronous exercise sessions, educational sessions, and remote telemonitoring. Telemonitoring encompassed three complementary functions: passive data collection (daily step count via smartwatch and symptom severity reporting), real-time physiological monitoring during exercise sessions (heart rate and SpO_2_ via the O2 ring, with individualized vibration alerts), and asynchronous clinical review of submitted data by the research team. Led by a registered physical therapist, participants attended two supervised exercise sessions per week (*n* = 16) via Zoom^®^. Groups of two to three participants were formed based on scheduling availability rather than functional capacity or oxygen requirement. Within each group, participants followed the same exercise sequence, but exercise dose, rest periods, and progression were individualized during each session. Each exercise session lasted approximately 45 min and included an initial check-in, warm-up, seated aerobic exercises, resistance exercises, cool-down, and final synchronization/check-out procedures (Table 1). Progression was generally planned as a 2-repetition-per-week increase in the aerobic and resistance tasks and was modified at the therapist’s discretion based on real-time assessment of dyspnea (Borg scale), SpO_2_, and heart rate during each session (Supplementary Materials Figure S1), as well as participant-reported symptoms between sessions. Initial exercise dose was determined individually based on each participant’s performance during the 1-min sit-to-stand test and 6-min walk test completed at the intake assessment. Participants receiving supplemental oxygen exercised according to their usual prescribed recommendations, and no adjustment to oxygen flow were made by the research team. Separate weekly education sessions (*n* = 8) on key topics were also delivered by a qualified healthcare professional. Each session consisted of approximately 30 to 40 min of presentation followed by 20–30 min of discussion and questions (Table 2).

#### Safety Procedures

When an O2 ring alert was triggered, participants were assessed by the therapist for severe dyspnea (Borg Scale ≥ 5) as the primary indicator, along with chest pain, dizziness, or lightheadedness, to determine whether the event was clinically significant or due to artifacts (e.g., device malfunction, disconnection, movement, or temperature variation). When the symptomatology correlated with the alert, the participant was instructed to stop exercise, rest in a seated position and initiate breathing control. If symptoms persisted beyond 5 min, structured interventions were added, including pursed-lip breathing and posture optimization (tripod position) with ongoing monitoring of SpO_2_ and symptom recovery trends. Exercise only resumed once SpO_2_ had returned to ≥88% and the participant demonstrated symptomatic recovery, defined as a reduction in dyspnea of at least one Borg category along with clinical stabilization.

The maximum recovery time after an interruption was predefined as 10–15 min of supervised recovery. If adequate physiological and symptomatic recovery was not achieved within this period, the session was terminated for that participant and recorded as an adverse or exercise intolerance event, prompting protocol adjustment. Failure to recover within 15 min, or the development of concerning symptoms (e.g., sustained severe dyspnea or chest pain), would have prompted clinician-directed escalation, including activation of emergency procedures and emergency medical services when indicated; however, these criteria were not met in the present study.

During exercise sessions, participants were instructed to wear both the smartwatch and the O2 ring to monitor heart rate and peripheral oxygen saturation (SpO_2_) and to avoid exceeding individualized target values. The smartwatch primarily served as a daily activity monitor for step count; the O2 ring was the primary device for real-time physiological monitoring during exercise sessions. Smartwatch data were not used to modify individual exercise prescriptions. Dyspnea was repeatedly assessed during the session using the Borg scale, with rest periods between exercise sections (Table 1). After each exercise session, participants synchronized the O2 ring with their smartphones or tablets and sent the data file to the research team by text or email. In parallel, participants were advised to wear the smartwatch daily to self-monitor their activity levels (step count), and to report symptom severity (dyspnea, fatigue, cough, sputum production, chest tightness, wheezing) through the Labfront Companion application. Symptom severity reports were used as part of patient self-monitoring and, when indicated, to guide modifications to the exercise protocol. Although daily use was encouraged throughout the 8-week intervention, synchronization of data from the smartwatch and symptom reporting was required only on exercise-session days. Participants could contact the therapist or research assistant at any time during the study for support, questions, or clarifications.

### 2.5. Outcomes

#### 2.5.1. Primary Outcomes

Feasibility outcomes included recruitment rate, study completion, dropout rate, session attendance, compliance with data acquisition and submission, compliance with symptom severity reporting, adverse events, and participant satisfaction. Recruitment rate was defined as the proportion of eligible individuals invited to the intake appointment who consented and attended the baseline assessment. Study completion was defined as the proportion of enrolled participants who completed at least 75% of the sessions and the final assessment, whereas dropout rate was defined as the proportion of enrolled participants who did not meet this threshold. Session attendance was calculated as the proportion of attended sessions out of the 24 scheduled sessions, including 16 supervised exercise sessions and 8 education sessions. Compliance with wearable-data acquisition and submission was calculated as the proportion of expected device-data submissions successfully received by the research team. Symptom severity reporting compliance was calculated as the proportion of expected symptom-severity reports submitted via the Labfront Companion application. Adverse events were defined as any undesirable event occurring during the intervention, such as COPD exacerbation, fall, musculoskeletal injury, severe dyspnea, pain, oxygen desaturation requiring interruption of exercise (beyond the maximum recovery time), medical emergency, or hospitalization. Participant satisfaction was assessed after the intervention using a brief feedback questionnaire developed by our team and previously used in a similar context [29] (Supplementary Materials Table S1). The questionnaire, not formally validated, was administered in printed format during the final in-person assessment. General comments expressed by the participants during the sessions were informally collected by the therapist and used to complement the participant satisfaction data.

#### 2.5.2. Secondary Outcomes

Exploratory clinical outcomes were assessed in-person (RespirabilityLab) during the intake appointment and at the end of the program. Those included lung function measured using a portable spirometer (Spirobank, MIR—Medical International Research S.p.A., Rome, Italy); dyspnea assessed with the modified Medical Research Council (mMRC) dyspnea scale [30] and the modified Borg scale (MBS) [31,32]; swallowing symptoms measured with the Sydney Swallow Questionnaire (SSQ) [33]; fatigue measured with the Fatigue Severity Scale (FSS) (total score and visual analog scale) [34]; self-efficacy measured with the Self-Efficacy for Managing Chronic Disease 6-Item Scale [35]; health-related quality of life measured with the Clinical COPD Questionnaire (CCQ) [36] and St George’s Respiratory Questionnaire (SGRQ) [37]; and functional exercise capacity assessed with the 1-min sit-to-stand test (1-min STST) test [38] and the 6-min walk test (6MWT) [39]. Predicted values for the 6MWT and 1-min STST were calculated using reference equations developed for healthy adults [40,41]. Higher scores indicate worse status for the mMRC, MBS, SSQ, FSS, CCQ, and SGRQ, whereas higher values indicate better status for the self-efficacy scale, 1-min STST, and 6MWT. Overall, these clinical outcomes were considered exploratory and were not used to determine intervention effectiveness.

### 2.6. Statistical Analysis

Descriptive statistics were used to summarize participant characteristics and study outcomes. Categorical variables were presented as counts and percentages, and continuous variables as mean ± standard deviation or median and interquartile range (IQR), as appropriate. Mann-Whitney U tests and Fisher’s exact tests were used to compare the characteristics of the participants who completed the intervention and those who did not. Pre-post changes in exploratory outcomes were analyzed using Wilcoxon signed-rank tests, and Cohen’s effect sizes were also calculated. All analyses were performed using IBM SPSS Statistics version 30.0 (IBM Corp., Armonk, NY, USA). Statistical significance was set at *p* < 0.05.

## 3. Results

### 3.1. Participant Flow and Baseline Characteristics

**Figure 1:**
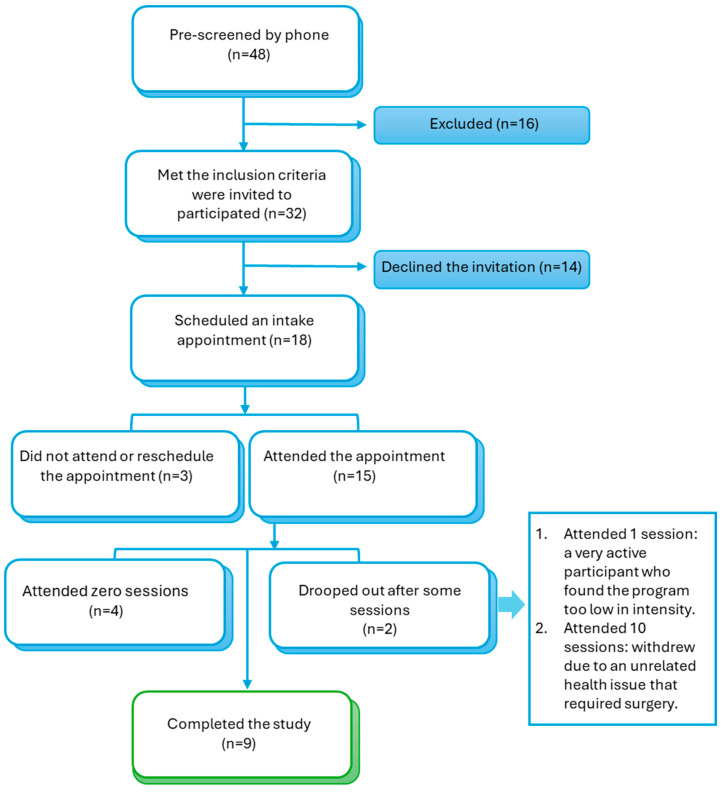
Participant flow chart.

Fifteen participants met the inclusion criteria, completed the intake assessment, and provided written informed consent; of these, nine completed the study (Figure 1). Participants who completed the study had significantly more years since COPD diagnosis and a lower percentage of predetermined FEV1/FVC values. No other statistically significant baseline differences were observed between groups (Supplementary Materials Table S2). The median age among completers was 67 years (IQR 62–73); seven (78%) were women (Table 3).

### 3.2. Primary Feasibility Outcomes

**Figure 2:**
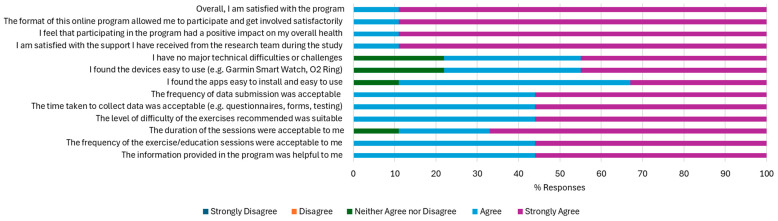
Participant feedback questionnaire.

Feasibility outcomes are presented according to the prespecified domains of recruitment, study completion, session attendance, compliance with data submission, occurrence of adverse effects, and participant satisfaction. Of the 32 eligible individuals invited to the intake appointment, 15 consented and attended the baseline assessment, resulting in a recruitment rate of 47% (calculated as the number of participants who attended the intake assessment divided by the number of eligible individuals contacted). Nine of the 15 enrolled participants completed at least 75% of the intervention sessions and the final assessment, yielding a study completion rate of 60% and a dropout rate of 40%. Mean session attendance was 94 ± 6.6%. Data submission rates averaged 86 ± 7.7% for the O2 ring and 97± 6.4% for symptom severity reporting, while smartwatch data were submitted by all participants (100%). No adverse events were reported during the intervention. Participant satisfaction was high among participants who completed the questionnaire, with eight participants strongly agreeing and one agreeing with the statements, “Overall, I am satisfied with the program” and “I feel that participating in the program had a positive impact on my overall health” (Figure 2).

Comments collected by the therapist during sessions also indicated generally positive perceptions of the intervention. Participants reported improved ability to perform physical activities across different levels, including meaningful gains in daily function such as standing independently and spending several hours at the mall for the first time in years. Improvements were also noted in recreational activities, such as golf performance, alongside increased self-confidence. One participant described reduced breathlessness over time, noting that early in the program, the O2 ring frequently triggered alerts during exercise, whereas these occurrences ceased later in the intervention. Some participants reported enjoying the intervention and expressed motivation to continue exercising following study completion. According to the lead therapist, challenges experienced during the program included occasional days of muscle soreness and mild symptom exacerbation requiring modifications to the exercise protocol for those individuals. These were considered expected intervention-related effects and did not meet the predefined criteria for adverse events (e.g., severe dyspnea, oxygen desaturation requiring exercise interruption, falls, or hospitalization). Minor technical issues related to device synchronization were also reported, but their frequency and impact on session delivery or data completeness were not systematically recorded. In addition, one participant experienced skin irritation associated with the watch. Despite these issues, real-time monitoring features of the O2 ring, particularly alerts for markedly low or high heart rate and SpO_2_, were considered valuable for supporting safety and clinical supervision during participation in the exercise program.

### 3.3. Secondary Exploratory Clinical Outcomes

No statistically significant pre-post changes were observed in the secondary exploratory clinical outcomes among participants who completed the study (Table 4). However, descriptively, the mean group distance increased by 29 m following the intervention. This change is within the reported minimal clinically important difference range (25–30 m) for this outcome [42], but should be interpreted as an exploratory signal given the small sample size and absence of statistical significance. Mean pre-post change in scores and 95% confidence intervals for all exploratory outcomes are provided in Supplementary Table S3.

## 4. Discussion

Findings indicated that an 8-week pulmonary telerehabilitation program delivered by videoconference and supported by commercial wearable telemonitoring devices was operationally deliverable among participants who completed the intervention, and no adverse events were observed in this small, selected group of participants. These findings are clinically relevant considering that access to PR remains limited despite its well-established benefits, and current ATS guidance now supports offering patients a choice between center-based PR and telerehabilitation. In that context, our results suggest that a supervised home-based model integrating exercise, education, and telemonitoring can be successfully implemented in adults with COPD within a real-world clinical setting [9,14].

The feasibility outcomes are broadly consistent with the growing telerehabilitation literature in COPD. The 2021 Cochrane review concluded that telerehabilitation can achieve outcomes comparable to center-based programs for chronic respiratory disease, while more recent syntheses have reinforced that home-based telehealth PR is generally similar to outpatient PR and superior to usual care for selected outcomes such as dyspnea and health status [16]. Importantly, Michaelchuk et al. noted substantial heterogeneity in program design and reporting, with relatively few studies using synchronous virtual supervision [43]. Our study adds to that literature by showing that a model based on synchronous group videoconferencing, weekly education, remote symptom severity reporting, and telemonitoring can be delivered with high adherence among those who remain engaged. These feasibility findings are also in line with earlier COPD studies, which reported that telerehabilitation was safe and viable, with no major adverse events and favorable patient acceptance [44].

Acceptability was another important indicator. Eight of nine completers strongly agreed that the program had a positive impact on overall health and that they were satisfied with it. This pattern is consistent with previous work showing high satisfaction with videoconference-based PR in COPD. In the qualitative extension study by Tsai et al., participants described positive virtual interactions, convenience, and perceived health benefits. At the same time, a broader systematic review of videoconferencing interventions in COPD also concluded that patient satisfaction was generally high even when technological issues arose [45]. Together, these data suggest that synchronous remote rehabilitation may be acceptable among selected participants who complete the intervention, which is critical for long-term implementation [46]. It should be noted that satisfaction was assessed at the end of the study and therefore only among completers, using a non-validated, non-anonymous questionnaire. This approach may have introduced social desirability bias and limits the generalizability of these findings to the broader enrolled population.

At the same time, participant retention remained a major challenge. Although adherence among completers was strong, the overall completion rate was 60%, similar to the 63% previously reported in the in-person publicly funded WRHA PR program [15]. Of the six participants who did not complete the program, four attended the intake assessment but did not initiate any sessions—two reported scheduling or time constraints, while reasons for the remaining two were not provided—and two withdrew after initiating: one after a single session due to perceived low exercise intensity, and one after ten sessions due to an unrelated health issue requiring surgery. Interestingly, participants who completed the in-person PR program had better baseline pulmonary function than those who did not complete it, whereas the opposite pattern was observed in the virtual PR program. These contrasting findings suggest that completion may be associated with distinct participant profiles across the two delivery models, supporting the potential complementarity of in-person and virtual PR in reaching a broader patient population. Similar completion rates suggest that remote PR delivery may mitigate certain participation barriers but does not substantially alter attrition patterns. Importantly, attendance, compliance, and satisfaction outcomes were derived exclusively from completers, which risks overestimating feasibility and acceptability for the broader enrolled population. Although telerehabilitation can improve geographical and logistical access, it does not eliminate other determinants of non-completion, such as disease burden, competing responsibilities, digital literacy, motivation, and caregiving constraints. In some cases, it also may introduce additional demands related to the use of devices and digital platforms. The withdrawal of one participant after a single session due to perceived low exercise intensity highlights the need for more flexible intensity levels in future program designs, particularly to accommodate patients with higher baseline functional capacity. Lewis et al. likewise observed that online PR was feasible and acceptable, but required substantial staff time, and that digital onboarding contributed importantly to engagement and safety [47]. Future studies should therefore prospectively collect reasons for dropout and document the relative contributions of clinical, technical, and contextual barriers [46].

No statistically significant pre-post changes were observed in the exploratory clinical outcomes, but this was not unexpected given the study’s feasibility focus and the small sample size. The more appropriate interpretation is not that the intervention lacked value, but that the study was not designed to provide a precise estimate of treatment effect. Nevertheless, numerical trends suggested a tendency toward improvement in dyspnea, self-efficacy, and 6-min walk test distance; these findings are hypothesis-generating only and should not be interpreted as evidence of effectiveness. The apparent discrepancy between the increase in absolute 6MWT distance and the unchanged median 6MWT % predicted should also be interpreted cautiously, as these values represent separate descriptive summaries in a very small sample, and the % predicted value was not calculated from the reported group median distance. These directional findings are consistent with prior COPD telerehabilitation studies that reported improvements in symptoms, self-efficacy, and exercise capacity after supervised remote programs. For example, the TeleR trial demonstrated improved self-efficacy and endurance exercise capacity compared with usual care [48]. At the same time, Marquis et al. reported significant gains in the 6MWT and symptom-related quality-of-life domains after 8 weeks of in-home telerehabilitation [49]. Larger comparative studies have also shown that supervised pulmonary tele-rehabilitation can produce outcomes comparable to those of center-based PR in severe COPD [19].

Important limitations should be considered when interpreting the results of this study. First, the sample size was small, no formal sample size calculation was performed, and the target enrollment was based on feasibility study methodology and available recruitment capacity; participants were recruited by convenience sampling, which limits external validity and increases the risk of selection bias. Furthermore, inclusion requirements regarding internet access, basic digital literacy, and availability of personal devices may limit generalizability to COPD populations with limited digital access or lower socioeconomic status. Second, the single-arm pre-post design without a comparator group precludes causal inference and does not allow the intervention’s effects to be distinguished from spontaneous variation, regression to the mean, or repeated-testing effects. Third, the dropout rate was substantial, and the exploratory analyses were limited to participants who completed the study, potentially introducing attrition bias and overestimating the feasibility of the intervention among the broader target population. Fourth, the study was not powered to detect clinically meaningful changes in patient-reported or functional outcomes. Fifth, formal screening for unstable cardiovascular disease, severe pulmonary hypertension, recent hospitalization, fall risk, and home environmental safety was not systematically conducted; additionally, formal criteria for modifying exercise progression were not predefined, relying instead on the therapist’s clinical judgment; and oxygen prescription flow rates for the two participants receiving supplemental oxygen therapy were not documented. Sixth, body composition variables such as fat-free mass and muscle mass were not assessed, which limits the interpretation of functional exercise capacity outcomes. Seventh, technical issues (including connectivity, synchronization, and missing data), as well as number of physiological alerts, exercise interruptions, and individual protocol modifications, were not systematically recorded by frequency or type. This limitation restricts our ability to report their impact on session delivery, clinical decision-making, and data completeness. Eighth, no a priori feasibility thresholds were established for recruitment, study completion, session attendance, or data submission compliance, which limits the interpretability of these outcomes. Future studies should predefine progression criteria for each feasibility domain to strengthen the interpretability of outcomes. Finally, although the feasibility results were encouraging, the findings should be interpreted as preliminary and specific to this implementation context.

This study draws on several important strengths that support the relevance of its findings. It evaluated a pragmatic intervention embedded in a real-world context, combined synchronous exercise supervision with telemonitoring and disease-specific education. The focus placed on implementation-relevant outcomes, including recruitment, attendance, data submission, safety, and patient satisfaction, enhanced its practical importance. By providing transparent documentation of session structure, supervision format, wearable integration, and compliance monitoring procedures, it contributes valuable operational information in an area characterized by considerable heterogeneity in program design and incomplete reporting [43]. The intervention model involved the integration of wearable telemonitoring within a supervised PR framework rather than using devices in isolation. Data submission from the smartwatch, O2 ring, and symptom severity reporting was high, supporting the practical feasibility of data capture and submission using consumer-grade devices in this context; however, these rates should not be interpreted as evidence of the clinical usefulness of telemonitoring by themselves. This is important since the wearable technology literature in COPD suggests that devices may be most useful when embedded within a broader rehabilitation or coaching model. In contrast, wearable-only strategies tend to yield smaller, less durable effects [23]. Shah et al. found modest improvements in step count and 6MWD, but limited impact on quality of life [23], and Coutu et al. emphasized that the transition from technological promise to routine care depends on workflow integration, clinical oversight, and governance considerations [21]. Our study supports that position by showing that wearables can function as part of a pragmatic tele-PR ecosystem centered on supervised exercise, symptom tracking, and clinician follow-up.

From clinical and research perspectives, these results support the continued development of remote PR pathways for people with COPD, especially where access to in-person programs is constrained, as recognized in current recommendations [9]. The next step should be a larger, methodologically stronger trial with predefined feasibility thresholds, explicit recording of reasons for withdrawal, standardized reporting of technical issues, and comparison against center-based PR or usual care. Future work should also clarify which patients are most likely to benefit from wearable-supported telerehabilitation, which monitoring signals are most actionable, and whether the addition of telemonitoring meaningfully improves adherence, safety, or outcomes beyond videoconference-based rehabilitation alone [21]. This research agenda is also consistent with prior work from Manitoba highlighting the value of PR and the need to expand accessible delivery models [15].

## 5. Conclusions

This study showed that an 8-week pulmonary telerehabilitation program delivered by videoconference and supported by telemonitoring devices was operationally deliverable among participants who remained engaged, with no adverse events observed in this small, selected group of participants with COPD. However, feasibility was limited by a substantial dropout rate, and findings apply only to a small, selected sample. Attendance, data submission, and participant satisfaction were high among participants who remained engaged with the program; however, these outcomes should be interpreted in the context of a substantial overall dropout rate. Although no statistically significant changes were observed in the exploratory clinical outcomes, some numerically favorable trends were identified, particularly in the 6MWT. Taken together, these findings support further development of telemonitoring-enabled telerehabilitation as a strategy to expand access to PR for people with COPD, while highlighting the need for larger controlled studies to confirm its effectiveness and better understand factors influencing retention and long-term implementation.

## Figures and Tables

**Table 1:** Structure of the Pulmonary Telerehabilitation exercise program.

Stage	Duration	Activity	Repetitions	Sets	Hold Time	Rest Time	Progression
**Chat & Check**	5 min	Chat and answer doubts	-	-	-	-	-
		O2 ring and Watch charged	-	-	-	-	-
		Fill the questionnaire (symptoms) using Labfront	-	-	-	-	-
		Remind to assess dyspnea (Borg scale, 0–10)	-	-	-	-	-
* At least 1 min of rest, assess and record dyspnea (Borg 0–10)							
**Warm-up**	5 min	Head turns	12	1 each side	2 s	10 s	-
		Chin-to-chest	12	1	2 s	10 s	-
		Seated quadratus lumborum (dynamic)	1	1 each side	10 s	10 s	-
		Posterior shoulder capsular	1	1 each side	10 s	10 s	-
		Triceps	1	1 each side	10 s	10 s	-
		Shoulder rolls (dynamic)	12	1 (forward and backward)	2 s	10 s	-
		Trunk rotation	12	1 each side	2 s	10 s	-
		Calves & Hamstrings (with blanket)	1	1 each side	10 s	10 s	-
* At least 1 min of rest, assess and record dyspnea (Borg 0–10)							
**Aerobic Exercises (seated)**	~10–15 min	Forward arm punches (dynamic)	10	1 each side	2 s	20–30 s	+2 reps/week
		Jumping jacks (diagonals) (dynamic)	10	1	2 s	20–30 s	+2 reps/week
		Stationary March with chair (dynamic)	20	2	2 s	20–30 s	+2 reps/week
* At least 1 min of rest, assess and record dyspnea (Borg 0–10)							
**Resistance exercises**	~15–20 min	Sit-to-stand	6	2	2 s	20–30 s	+2 reps/week
		Heel raises	6	2	2 s	20–30 s	+2 reps/week
		Biceps curls (food can as weight)	6	2	2 s	20–30 s	+2 reps/week
		Side and front arm raises (food can as weight)	6	2	2 s	20–30 s	+2 reps/week
		Triceps (food can as weight)	6	2	2 s	20–30 s	+2 reps/week
		Bicycle crunch (diagonal)	6	2	2 s	20–30 s	+2 reps/week
* At least 1 min of rest, assess and record dyspnea (Borg 0–10)							
**Cool down**	5 min	Neck stretch	1	1 each side	10 s	10 s	-
		Wrist rotation	10 (5 right/5 left)	1 each side	10 s	10 s	-
		Wrist flexor/extension	1	1 each side	10 s	10 s	-
		Chest	1	1	10 s	10 s	-
		Seated quadratus lumborum (dynamic)	1	1 each side	10 s	10 s	-
		Seated leg stretches	2	1 each side	10 s	10 s	-
* At least 1 min of rest, assess and record dyspnea (Borg 0–10)							
**Chat & Sync**	5 min	Chat and answer doubts	-	-	-	-	-
		Synchronize O2 ring to forward info	-	-	-	-	-
		Reminder to synchronize the watch later at night	-	-	-	-	-

* Rest periods between sections were at least one minute, extended at the therapist’s discretion based on real-time dyspnea, heart rate, and SpO_2_ values.

**Table 2:** Education sessions.

Session	Topics	Delivered by
1	Intro to Pulmonary Rehab	Respiratory Therapy
2	Managing shortness of breath	Respiratory Therapy
3	Exercising well	Physical Therapist
4	Risk factors, Triggers, and Action plan	Respiratory Therapy
5	Respiratory medication & Medication delivery services	Pharmacist
6	Healthy eating	Physical Therapist
7	Energy conservation and Management of Fatigue and Stress	Physical Therapist
8	Dysphagia	Speech Pathologist

Each session consisted of 30 to 40 min presentation followed by 20 to 30 min of Q&A.

**Table 3:** Baseline characteristics of the participants.

	*n* = 9
**Demographics**	
Sex, Female/Male	7/2
Age (years)	67 (62–73)
Height (cm)	168 (164–171)
Weight (kg)	73 (55–101)
BMI (kg/m^2^)	25 (20–36)
Time since COPD diagnosis (years)	10 (6–17)
**Pulmonary function**	
Lung function (% pred)	
FVC	75 (69–83)
FEV1	55 (42–67)
FEV1/FVC	67 (60–89)
Peak expiratory flow	66 (49–95)
**Symptoms**	
Modified Medical Research Council dyspnea scale (0–4)	1 (1–2)
Modified Borg scale (0–10)	3 (1–4)
Sydney Swallow Questionnaire ≥ 180, *n* (%)	5 (56)
Fatigue Severity Scale	
Total score	3.7 (2.7–5.8)
Visual Analog Scale	5 (4–9)
Symptom severity (0–10)	
Dyspnea	2.1 (1.8–2.9)
Fatigue	2.8 (2.4–3.1)
Cough	2.1 (1.8–2.1)
Sputum production	1.9 (1.6–2.4)
Chest tightness	2.2 (1.7–3.1)
Wheezing	1.4 (1.1–1.5)
**Functional outcomes**	
Self-Efficacy for Managing Chronic Disease 6-Item Scale (1–10)	6.3 (5.3–8.1)
1-min STST (repetitions)	22 (20–26)
1-min STST (% predicted)	69 (60–75)
6MWT (meters)	378 (365–468)
6MWT (% predicted)	89 (66–100)
Resting SpO_2_ (%)	97 (94–98)
SpO_2_ nadir post-6MWT (%)	94 (94–97)
Resting HR (bpm)	82 (71–87)
Peak HR post-6MWT (bpm)	93 (67–108)
Daily steps	4331 (3028–5571)
**Quality-of-life outcomes**	
Clinical COPD Questionnaire (0–6)	
Symptoms	2.5 (1.6–2.8)
Functional	1.5 (0.8–2.6)
Mental State	2.5 (0.5–4.5)
Total	6.3 (5.3–8.1)
St George’s Respiratory Questionnaire (0–100)	
Symptoms	59.3 (37.8–72.1)
Activity	79.8 (61.4–100)
Impact	27.3 (13.8–46.9)
Total	52.6 (29.2–65.9)

Abbreviations: COPD: Chronic Obstructive Pulmonary Disease; FEV1: Forced expiratory volume in the first second; FVC: Forced vital capacity; FSS: Fatigue severity scale; HR: Heart rate; SpO_2_: Peripheral oxygen saturation; 1-min STST: 1-min sit-to-stand test; 6MWT: 6-min walk test. Median (IQR) is reported unless otherwise specified.

**Table 4:** Participants outcomes pre and post intervention (*n* = 9).

	Median (IQR)			
Outcomes	Pre	Post	*p*-Value	Effect Size (r)
Modified Medical Research Council dyspnea scale (0–4)	1.0 (1.0–2.0)	1.0 (1.0–1.5)	0.16	0.47
Modified Borg scale (0–10)	3.0 (1.0–3.8)	2.0 (1.0–3.0)	0.27	0.37
FSS				
Total score	3.7 (2.7–5.8)	5.1 (3.6–6.0)	0.14	0.49
Visual Analog Scale	5.0 (4.0–9.0)	5.0 (2.0–7.0)	0.34	0.32
Self-Efficacy for Managing Chronic Disease 6-Item Scale (1–10)	6.3 (5.3–8.1)	7.5 (6.0–8.2)	0.12	0.51
Clinical COPD Questionnaire (0–6)				
Symptoms	2.5 (1.6–2.8)	3.3 (2.3–3.6)	0.06	0.63
Functional	1.5 (0.8–2.6)	1.5 (1.4–2.4)	0.23	0.40
Mental State	2.5 (0.5–4.5)	2.5 (0.8–3.0)	0.67	0.14
Total	2.0 (1.3–3.0)	2.4 (2.2–2.7)	0.51	0.22
St George’s Respiratory Questionnaire (0–100)				
Symptoms	59.3 (37.8–72.1)	62.6 (33.5–74.8)	0.76	0.10
Activity	79.8 (61.4–100.0)	79.2 (49.2–100.0)	0.69	0.14
Impact	27.3 (13.8–46.9)	26.5 (13.3–42.8)	0.59	0.18
Total	52.6 (29.1–65.9)	50.5 (27.7–64.3)	0.44	0.26
1-min STST (repetitions)	22.0 (19.5–25.5)	21 (19.0–25.5)	0.23	0.50
1-min STST (% predicted)	69.2 (59.8–75.4)	70.6 (53.4–79.2)	0.23	0.50
6-MWT (meters)	378.0 (364.0–467.8)	475.0 (402.0–485.5)	0.21	0.42
6-MWT (% predicted)	89.2 (65.7–100.9)	89.2 (77.1–110.7)	0.33	0.33

Abbreviations: COPD: Chronic Obstructive Pulmonary Disease; FSS: Fatigue severity scale; 1-min STST: 1-min sit-to-stand test; 6MWT: 6-min walk test. Wilcoxon signed-rank test. Cohen’s r = small ≥ 0.10, moderate ≥ 0.30, and large ≥ 0.50. Note: The unchanged median 6MWT % predicted reflects that the participant occupying the median position for this variable remained the same before and after the intervention, independent of the shift in median absolute distance.

## Data Availability

The data presented in this study are available from the corresponding author upon reasonable request, in accordance with ethical restrictions.

## Supplementary Materials

The following supporting information can be downloaded at: https://www.mdpi.com/article/10.3390/jcm15135292/s1, Figure S1: Participant perceived dyspnea and physiological responses during exercise, Table S1: Study Participant Feedback Questionnaire, Table S2: Comparison of participant characteristics according to study completion status, Table S3: Pre–post intervention change in scores.
