# Supplementary material for: Pulmonary Telerehabilitation and Telemonitoring for Patients with Chronic Obstructive Pulmonary Disease: A Single-Arm Pilot Feasibility Study
Source: J Clin Med. 2026 Jul 7;15(13):5292. doi: 10.3390/jcm15135292 (PMC13362692; doi:10.3390/jcm15135292)
Supplement: Supplementary file 1 [file jcm-15-05292-s001.zip › jcm-4371189-supplementary.pdf]

Supplementary material
Figure S1. Participant perceived dyspnea and physiological responses during exercise

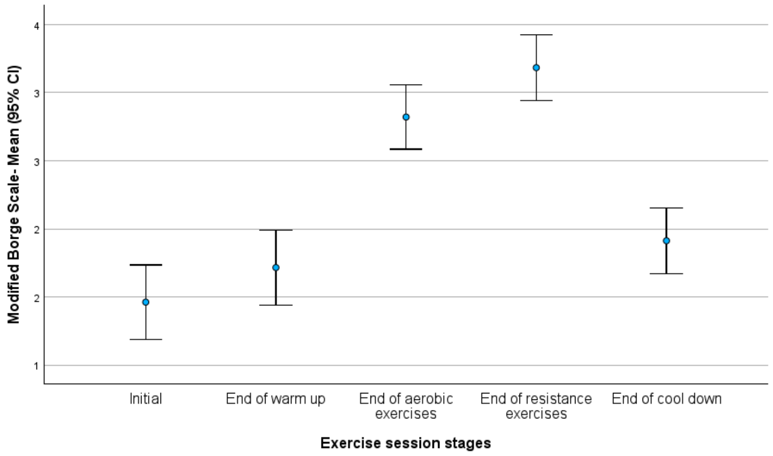

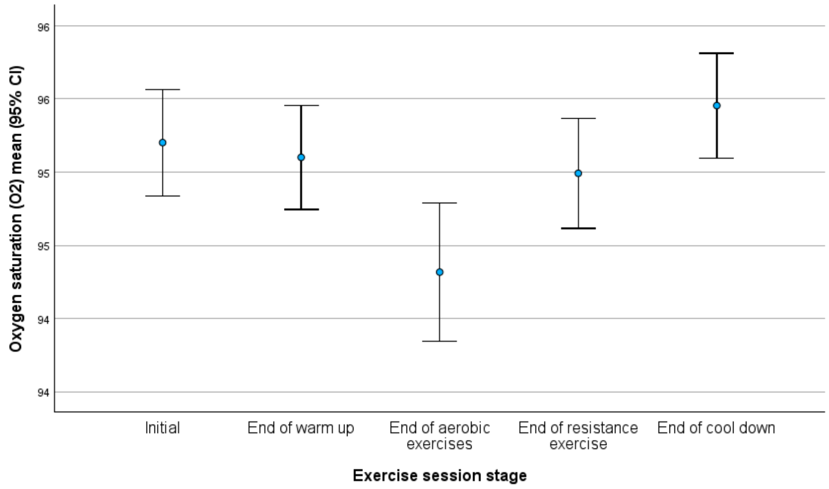

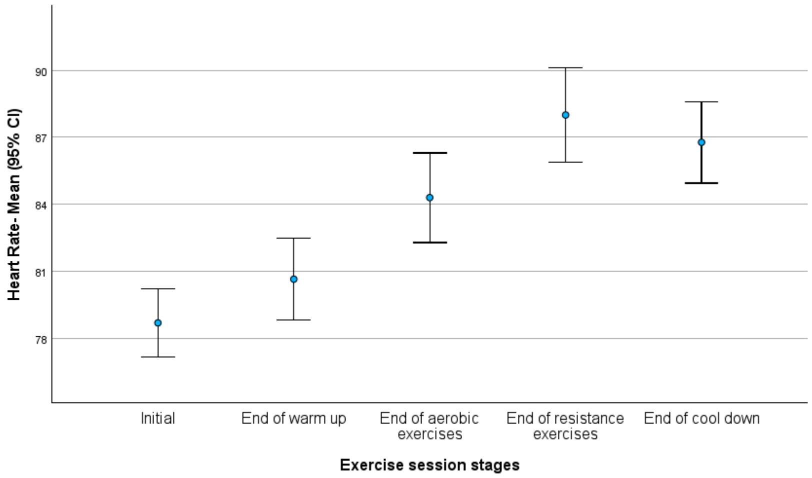

Table S1. Study Participant Feedback Questionnaire (SPFQ)

Thank you for your participation. Your experiences in this study are important to us and we would like to hear about them. Your answers will help us improve future studies, and there are no right or wrong answers.

It will take approximately 5-10 minutes to complete.

Please select one response for each of the following items below	**Strongly Disagree**	**Disagree**	**Neither Agree nor Disagree**	**Agree**	**Strongly Agree**
1. The information/education provided in the program was helpful to me	0	1	2	3	4
2. The frequency of the exercise/education sessions were acceptable to me (3x a week, 8 weeks)	0	1	2	3	4
3. The duration of the sessions were acceptable to me (30-45min)	0	1	2	3	4
4. The level of difficulty of the exercises/education recommended was suitable	0	1	2	3	4
5. The time taken to collect data was acceptable (e.g. questionnaires, forms, testing)	0	1	2	3	4
6. The frequency of data submission was acceptably (e.g. diary 3x a week)	0	1	2	3	4
7. I found the apps easy to install and easy to use	0	1	2	3	4
8. I found the devices easy to use (e.g. Garmin Smart Watch, O2 Ring)	0	1	2	3	4
9. I have no major technical difficulties or challenges	0	1	2	3	4

10. I am satisfied with the support I have received from the research team during the study	0	1	2	3	4
11. I feel that participating in the program had a positive impact on my overall health	0	1	2	3	4
12. The format of this online program allowed me to participate and get involved satisfactory	0	1	2	3	4
13. Overall, I am satisfied with the program	0	1	2	3	4

Table S2. Comparison of participant characteristics according to study completion status

Baseline characteristics of the participants			
	Completed the study		
	No (n=6)	Yes (n=9)	*p*-value
Sex n (%)			
Female	6 (100)	7 (78)	0.49
Age (years)	66 (61-70)	67 (62-73)	0.86
Time since COPD diagnosis (years)	4 (2-7)	10 (6-17)	0.02
Lung function (%pred)			
FVC	96 (61-99)	75 (69-83)	0.48
FEV1	92 (43-106)	55 (42-67)	0.20
FEV1/FVC	98 (74-106)	67 (60-89)	0.04
Peak expiratory flow	102 (68-125)	66 (49-95)	0.16
Modified Medical Research Council dyspnea scale (0-4)	2 (0-3)	1 (1-2)	1.00
Modified Borg scale (0-10)	1 (0-3)	3 (1-4)	0.26
Sydney Swallow Questionnaire ≥ 200 n (%)	1 (17)	5 (56)	0.29
FSS			
Total score	4.4 (1.6-6.6)	3.7 (2.7-5.8)	1.00
Visual Analog Scale	8 (4-9)	5 (4-9)	0.81
Self-Efficacy for Managing Chronic Disease 6-Item Scale (1-10)	7.0 (4.8-9.1)	2.0 (1.3-3.0)	0.63
Clinical COPD Questionnaire (0-6)			
Symptoms	1.9 (1.4-3.3)	2.5 (1.6-2.8)	0.90
Functional	1.5 (0.2-3.8)	1.5 (0.8-2.6)	0.91
Mental State	1.8 (0.4-3.1)	2.5 (0.5-4.5)	0.44
Total	1.9 (0.7-3.1)	6.3 (5.3-8.1)	0.68
St George's Respiratory Questionnaire (0-100)			
Symptoms	49.4 (17.4-54.3)	59.3 (37.8-72.1)	0.16
Activity	65.1 (13.4-100)	79.8 (61.4-100)	0.76
Impact	20.9 (0-64.3)	27.3 (13.8-46.9)	0.77
Total	36.3 (10.4-74.4)	52.6 (29.2-65.9)	0.68
1-min STST (repetitions)	20 (14-38)	22.5 (20.3-26.3)	0.75
1-min STST (% predicted)	33 (32-35)	33.5 (30.8-34.8)	0.79
6MWT (meters)	378 (293-507)	378 (365-468)	0.81
6MWT (% predicted)	81 (69-96)	89 (66-100)	0.91

Abbreviations: COPD: Chronic Obstructive Pulmonary Disease; FEV1: Forced expiratory volume in the first second; FVC: Forced vital capacity; FSS: Fatigue severity scale;1-min STST: 1-minute sit-to-stand test; 6MWT: 6-minute walk test. Median (IQR25-75%) are reported unless otherwise specified. Mann-Whitney U test for continuous outcomes; Fisher's exact test for binary outcomes

Table S3. Pre–post intervention changes in scores (n = 9)

Outcome	Mean change (post − pre)	SD	95% CI
Symptoms			
mMRC dyspnea scale (0–4)	−0.22	0.44	−0.56, 0.12
Modified Borg scale (0–10)	−0.72	1.80	−2.11, 0.66
Fatigue			
FSS Total score	+2.92	7.72	−3.01, 8.86
FSS Visual Analog Scale	−1.56	3.40	−4.17, 1.05
Self-efficacy			
Self-Efficacy for Managing Chronic Disease (1–10)	+0.71	1.21	−0.23, 1.64
Clinical COPD Questionnaire (0–6)			
Symptoms	+0.72	0.93	0.01, 1.44
Functional	+0.19	0.94	−0.53, 0.92
Mental State	−0.44	1.86	−1.88, 0.98
Total	+0.28	0.89	−0.41, 0.96
St George's Respiratory Questionnaire (0–100)			
Symptoms	−0.18	12.14	−9.51, 9.15
Activity	−1.98	13.03	−12.00, 8.04
Impact	−2.67	9.19	−9.73, 4.40
Total	−1.70	6.38	−6.60, 3.20
Functional outcomes			
1-min STST (repetitions)	+0.67	3.71	−2.18, 3.52
1-min STST (% predicted)	+2.32	10.62	−5.84, 10.48
6MWT (meters)	+28.94	72.12	−26.50, 84.38
6MWT (% predicted)	+6.13	15.02	−5.41, 17.68

Note. Mean change = post-intervention minus pre-intervention value. Positive values indicate increase; negative values indicate decrease. All findings are exploratory and should not be interpreted as evidence of intervention effectiveness. CI = confidence interval; FSS = Fatigue Severity Scale; mMRC = modified Medical Research Council; SD = standard deviation; STST = sit-to-stand test; 6MWT = 6-minute walk test.
